# Oral Rehabilitation of a Pediatric Patient with Vitamin D-dependent Rickets II: A Rare Case Report

**DOI:** 10.5005/jp-journals-10005-1586

**Published:** 2019

**Authors:** Anchal Chhonkar, Anil Gupta, Payal Chaudhary, Vani Kapoor

**Affiliations:** 1,3,4 Department of Pedodontics and Preventive Dentistry, SGT Dental College Hospital and Research Institute, Gurugram, Haryana, India; 2 Department of Pedodontics and Preventive Dentistry, SGT University, Gurugram, Haryana, India

**Keywords:** Hypoplastic teeth, Recurrent spontaneous abscess, Vitamin D-dependent rickets

## Abstract

Vitamin D-dependent rickets (VDDR) is a disorder of bone development characterized by softened weak bones. It is of two types–vitamin D-dependent rickets-I (VDDR-I) and vitamin D-dependent rickets type II (VDDR-II).

Vitamin D-dependent rickets type II is a rare autosomal recessive disorder. The patients exhibit characteristic clinical features as well as numerous dental manifestations such as hypoplastic teeth, missing teeth, enlarged pulp chambers, and recurrent spontaneous abscesses.

The case presented in this article is of vitamin D-dependent rickets type II in a pediatric dental patient who reported with multiple missing teeth and required treatment consisting of restorations and extractions. The aim of the present article is to raise awareness on the characteristics of this disorder, as well as its treatment and dental considerations.

**How to cite this article:**

Chhonkar A, Gupta A, *et al.* Oral Rehabilitation of a Pediatric Patient with Vitamin D-dependent Rickets II: A Rare Case Report. Int J Clin Pediatr Dent 2019;12(1):73–75.

## INTRODUCTION

Vitamin D-dependent rickets (VDDR) is a disorder of bone development characterized by softened weak bones.^1^ It is of two types—vitamin D-dependent rickets-I (VDDR-I) and vitamin D-dependent rickets type II (VDDR-II).^1,2^

VDDR-I also termed as pseudovitamin D resistant rickets is less severe than VDDR-II.^3^ VDDR-II results from the end organ resistance to active metabolite 1,25‐dihydroxyvitamin D (1,25(OH)_2_ D). The prevalence rate of VDDR-II is usually reported to be around 1:20,000.^4,5^ VDDR-II is also termed as hypophosphatemic vitamin-D resistant rickets.^6^ It is characterized by persistent hypophosphatemia and hyperphosphaturea and is associated with decreased renal tubular reabsorption of inorganic phosphates.^4^

It also includes hypocalcemia, secondary hypothyroidism, rickets and also partial or full alopecia.^3^ Dental manifestations include poor calcification of the alveolar bone, fracture and attrition of enamel, hypoplastic dentin. Recurrent formation of spontaneous abscesses is a common feature observed in these patients.^7^ These affect multiple caries or trauma to free primary or permanent teeth and are related to a deficient dentin mineralization.^8^

## CASE REPORT

A 6-year-old male patient reported to the outpatient department of Department of Pedodontics and Preventive Dentistry, Faculty of Dental Sciences, SGT University, Bhudera, Gurugram, with a chief complaint of missing upper and lower front teeth since the past two years. The patient also experienced sudden sharp shooting pain on mastication in the left lower back tooth since the past one month. The patient also gave history of repeated injuries due to fall. Three years back, the patient had been diagnosed with rickets, specifically VDDR II. He had also been hospitalized twice before due to his inability to walk. His family reported that the patient had never been immunized. This was his first dental visit.

**Figs 1A to C:**
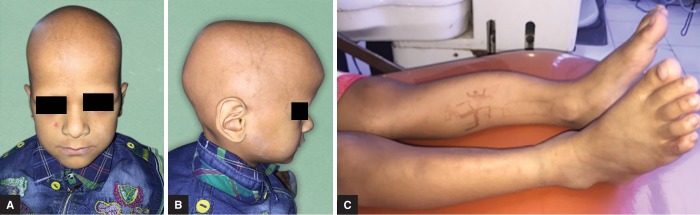
Extraoral examination findings revealed (A) Alopecia; (B) Frontal bossing; (C) Bowing of legs

On extraoral examination, a bruise was observed on the right cheek which had occurred due to a fall two days back. Characteristic signs of rickets were observed such as—alopecia, frontal bossing, and bowing of legs (Fig. 1).

**Figs 2A and B:**
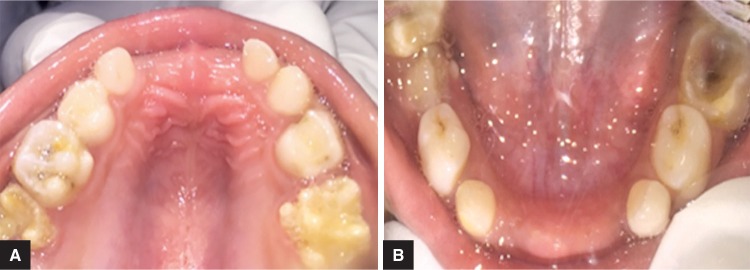
(A) Clinically missing 51, 52, 61, 65. Hypoplastic 16,26; (B) Clinically missing 71, 72, 81, 82. Grossly decayed 75

On intraoral examination, clinically missing 51, 52, 61, 65, 71, 72, 81, 82 were observed. Decay was present in 55, and 75 was grossly decayed. All the permanent first molars were found to be hypoplastic (Fig. 2).

**Fig. 3:**
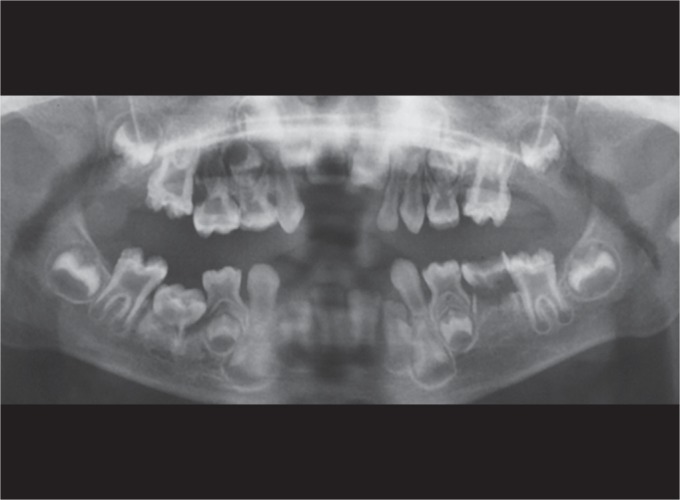
Au OPG shows no evidence of tooth buds of the second premolars

His orthopantomogram (OPG) findings revealed absence of permanent tooth buds of all four second premolars. Resorbed roots were observed in 75. Large pulp chambers were observed with thin overlying enamel and dentin (Fig. 3).

### Treatment Plan

**Figs 4A and B:**
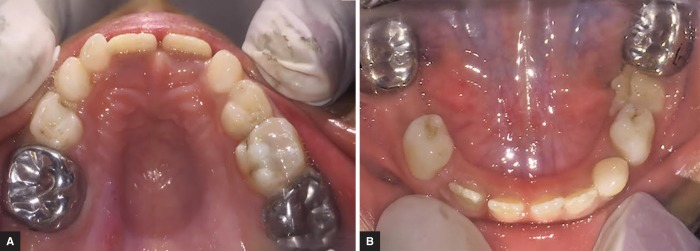
Postoperative photographs showing restorations and stainless steel crowns in upper (A) and lower (B) arches

On the first visit, the patient along with his family was counselled about the proper diet, oral hygiene measures, and preventive measures. Restorations of the carious teeth and extraction of the left mandibular primary molar under local anesthesia were conducted in the subsequent visits. Stainless steel crowns were then given on the hypoplastic permanent molars. The patient is currently on follow up and we await the mesial shift of the left mandibular permanent first molars (Fig. 4).

## DISCUSSION

Vitamin D-dependent rickets-II is a rare autosomal recessive disorder. In India, VDDR-I is more common whereas in developed countries the more common one is VDDR-II. The term ‘Vitamin D Resistant Rickets’ was first used in 1937 by Albright et al.^3^ VDDR-II is also known as familial hypophosphatemia, vitamin D refractory rickets, and phosphate diabetes.^8^ The cause of VDDR-II is vitamin D receptor mutation. It results from a selective disorder of transepithelial transport of phosphate in the kidney that leads to decreased tubular reabsorption of phosphate and persistant hypophosphatemia. The low level of serum phosphate leads to rickets.^8^ This disorder is more common in the Fanconi syndrome.^9^

In our patient, the left mandibular primary second molar was grossly decayed and after clinical and radiographic evaluation we decided to extract it. The right maxillary second primary molar exhibited occlusal caries and was restored. It is imperative to prevent the spread of infection to pulp in such teeth, since it would lead to the formation of spontaneous recurrent abscesses by bacteria invading through enamel cracks and dentinal microcleavages.

Patients with VDDR-II present with primary and permanent teeth having dentinal dysplasia,^10^ as evident in our patient as well. The patient had clinically missing permanent maxillary and mandibular incisors, since he is six years old, we have kept him on follow up to observe the time of eruption of these teeth. Delayed dentition is found in 47% of patients with VDDR-II.

On radiographic examination of the patient, we observed teeth having thin enamel and dentin layers. Abe et al. suggested that globular dentin caused by hypophosphatemia impairs calcification. The incompletely mineralized dentin exists in the form of calcospherites, which trap microorganisms and also impede mechanical endodontic cleaning. Also, the thin dentin layer perforates easily and does not support restorative posts for prosthetic crowns.^11^ Hillmann and Geurtsen found that the permanent teeth might also be affected and histopathologic examination of the permanent dentition is necessary.^12^

In our patient, we adapted and cemented stainless steel crowns on all four permanent first molars. Prophylactic coverage of these teeth with stainless steel crowns on molars and composite resin on the anterior teeth should be done with caution. The crown preparation should be minimal to avoid inadvertent pulp exposure. Another critical factor is the loss of vertical dimensions, if multiple posterior primary teeth need to be extracted. Thus, there is a delicate balance between the benefits and possible risks of using stainless steel crowns.

Pit and fissure sealants are useful when the teeth are erupting as they prevent ingress of bacteria into the enamel microfractures as well as initiation of caries in the deep pits and fissures.^13^

## CONCLUSION

Patients with VDDR-II show some peculiar dental abnormalities. In our study, dental caries as common as delay in dentition were the most prevalent problems in VDDR-II patients that could be reduced by proper dental care and good oral hygiene. So, the dentist as well as pediatrician should be made aware of the features of disorder and early intervention.
